# B7-H3 promotes aggression and invasion of hepatocellular carcinoma by targeting epithelial-to-mesenchymal transition via JAK2/STAT3/Slug signaling pathway

**DOI:** 10.1186/s12935-015-0195-z

**Published:** 2015-04-21

**Authors:** Fu-biao Kang, Ling Wang, Heng-chuan Jia, Dong Li, Hai-jun Li, Yin-ge Zhang, Dian-xing Sun

**Affiliations:** Department of Liver Diseases, Bethune International Peace Hospital, Shijiazhuang, Hebei People’s Republic of China; Chinese PLA Medical School, Chinese PLA General Hospital, Beijing, People’s Republic of China; Cancer Research Institute, the Fourth Hospital of Hebei Medical University, Shijiazhuang, Hebei People’s Republic of China

**Keywords:** Hepatocellular carcinoma, B7-H3, Invasion, Epithelial-To-Mesenchymal Transition, JAK/STAT signaling pathway

## Abstract

**Background:**

B7-homologue 3 (B7-H3), a recently identified immunoregulatory protein, has been shown to be overexpressed in human hepatocellular carcinoma (HCC). However, whether the dynamic expression pattern of B7-H3 contributes to early invasion of HCC is largely unknown. In addition, the biological roles of B7-H3 in HCC are still unclear. Herein, we are going to examine B7-H3 expression profile and its clinicopathological significance in primary and metastatic HCC, and further determine whether B7-H3 knockdown simulates different pathological states of HCC progression and metastasis.

**Methods:**

Using immunohistochemistry, B7-H3 expression was studied on 116 HCC containing primary and metastatic HCCs. Survival curves and log-rank tests were used to test the association of B7-H3 expression with survival. HCC cells with B7-H3 depletion were established by RNA interference to investigate the effect of B7-H3 on cell proliferation, apoptosis, migration and invasion in vitro.

**Results:**

Statistical analysis of clinical cases revealed that B7-H3 high expression group had inclinations towards late TNM stage, the presence of vascular invasion, lymph metastasis, and the formation of microsatellite tumors. Increased intensity of tumor B7-H3 staining was detected more significantly in metastatic HCC tumors. Consistently in experiments performed in vitro, B7-H3 was able to stimulate the wound healing, metastasis and invasion of hepatoma cells by targeting epithelial-to-mesenchymal transition (EMT) via JAK2/Stat3/Slug signaling pathway, while no obvious influence on cell growth and apoptosis.

**Conclusion:**

B7-H3 in the regulation of the metastatic capacity of HCC cells makes itself a promising therapeutic target for anti-metastasis therapy.

**Electronic supplementary material:**

The online version of this article (doi:10.1186/s12935-015-0195-z) contains supplementary material, which is available to authorized users.

## Introduction

Hepatocellular carcinoma (HCC) is the fifth most frequent cancer in the world, and affects more than 1 million people annually worldwide with a mortality rate almost equal to its incidence [1]. Although great advances in surgical technique and medical care have been achieved over the last several decades, the 5-year survival rate worldwide of HCC is still less than 5%, mainly because of the high rate of recurrence and metastasis [2]. It would be clinically beneficial to discover biological markers that help predict early tumor metastasis, identify their molecular mechanisms invasion processes of HCC patients, and then design a better therapeutic target for early intervention of cancer invasion and metastasis. In fact, increasing evidence suggests that HCC metastasis is a multistep process including tumor cells’ invasion into the extracellular matrix and stromal cell layers, intravasating into the lumina of blood vessels, extravasating into the parenchyma of distant organs and then invading foreign microenvironments in order to form micrometastasis [3-5].

B7-H3 (B7 homologue 3), a newly found member of B7/CD28 superfamily, was identified as an accessory costimulatory molecule after initial antigen priming in cooperation with a putative counterreceptor [6-9]. The role of B7-H3 in adaptive immune responses still remains controversial. B7-H3 was first identified as a costimulatory molecule that engages its receptor on T cells to promote T-cell activation and secretion of IFN-γ [6,10]. Others evidence showed that B7-H3 played an inhibitory role during autoimmune diseases [11,12]. In tumor, B7-H3 has been described in a number of different tumor entities, for example, in prostate, pancreatic and renal cell cancer and neuroblastoma [7,8,13,14]. Recently, Sun et al. showed that B7-H3 was abundantly expressed in HCC and involved in evading anti-tumor immunity, but the specific mechanism was still elusive [15]. As a tumor-associated antigen, B7-H3 has not only been attributed to its involvement in tumor immunity but also plays a non-immunological role in cancer progression [9]. In 2008, Chen et al. showed for the first time that downregulation of B7-H3 reduced cell adhesion to fibronectin by up to 50% and migration and invasion by more than 70% in melanoma and breast cancer cells [16]. In agreement with this, Yuan et al. showed that B7-H3 downregulation led to significant inhibition of cell migration and invasion of human prostate cancer cells [13]. These results strongly suggest that B7-H3 was involved in cancer progression and metastasis beyond modulating tumor immunity.

In this present study, we carried out the immunohistochemistry study to characterize the B7-H3 expression in human HCC tissues and found a positive correlation with metastasis of HCC. In addition, we have found that downregulation of B7-H3 could reduce cell migration and matrigel-invasion significantly in HCC cells, but surprisingly had no apparent impact on cell proliferation. These results could be attributed to the functions of B7-H3 in the regulation of epithelial to mesenchymal transition (EMT) process via JAK2/STAT3/Slug pathway. In conclusion, our data indicate B7-H3 as a readily-detectable biomarker for tumor recurrence and/or metastasis and suggest an attractive target of B7-H3 for therapeutic manipulation in the future for multimodal management of HCC.

## Methods

### Patients and clinical specimens

According to the following the inclusion and exclusion criteria, 116 HCC tumor tissues and corresponding adjacent noncancerous liver tissues used in immunohistochemical analysis were randomly obtained from patients undergoing liver curative resection between 2004 and 2008 hospitalized in department of hepatobiliary surgery, Bethune International Peace Hospital and the Fourth Hospital of Hebei Medical University. (a) distinctive pathologic diagnosis of HCC, (b) without anticancer treatment before liver resection, (c) underwent primary and curative resection for HCC between 2004 and 2008, and (d) complete clinicopathologic and follow-up data. 20 normal liver tissues were also taken from the biopsy tissues of healthy living donors for transplantation. All specimens were collected in the operating theater immediately (≤15 min) after resection of the tumors and then were snap frozen in liquid nitrogen or fixed in 10% buffered formalin solution and embedded in paraffin for histological analysis. The histologic grade of tumor differentiation was determined by the Edmondson-Steiner grading system. Liver function was assessed by the Child-Pugh score system. Tumors were classified according to the WHO classification and the International Union against Cancer tumor-node-metastasis (TNM) classification. If patients had multiple lesions in the liver, we selected the main nodule for our study. All samples were obtained with informed consent and their use was approved by the ethics committee of the institution. The median follow-up period was 33.5 months (range, 9–62 months; standard deviation [SD], 11.6 months). At the last follow-up (Dec 31st, 2012), 79 (68.1%) patients finally died, including 32 due to liver failure or bleeding from the gastrointestinal tract and the remaining 47 cases due to tumor recurrence.

### Cell lines and culture conditions

Human HCC cell lines (HepG2 and SMCC-7721) were obtained from the Cell Bank of the Chinese Academy of Sciences (Shanghai, China) and cultured according to the instructions from American Type Culture Collection (ATCC). The cells were maintained in high-glucose DMEM (Gibco) supplemented with 10% fetal calf serum (Gibco). The cells were incubated at 37°C in a humidified chamber containing 5% CO_2_.

### Antibodies

Goat anti-B7-H3 was purchased from R&D company for immunohistochemistry. B7-H3 monoclonal antibody (Biolegend MIH35) was used in vitro experiment. Rabbit anti-E-cadherin, anti-N-cadherin, anti-Vimentin, anti-Slug, and anti-Snail antibodies were purchased from Abcam company. Rabbit anti-phospho-Stat3 (Tyr705), Stat3, Phospho-Jak2 (Tyr1008), and Jak2 were purchased from Cell Signalling Technology. Anti-GAPDH antibody was purchased from Santa Cruz Biotechnology.

### Immunohistochemical staining

Immunohistochemistry was performed using the Dako EnVision™ method according to the manufacturer’s instructions. In brief, 4-μm thick consecutive sections were cut by microtome, dewaxed in xylene and rehydrated through graded ethanol solutions. Antigens were retrieved by heating the tissue sections at 100°C for 30 min in EDTA solution. Sections were cooled down and immersed in 0.3% H_2_O_2_ solution for 20 min to block endogenous peroxidase activity, and then rinsed in PBS for 5 min, blocked with 5% BSA at room temperature for 20 min, and incubated with primary antibodies against B7-H3 (final concentration in use, 5 μg/ml) at 4°C overnight. Negative controls were performed by replacing the specific primary antibody with PBS. After three PBS washes, sections were incubated with secondary antibodies for 30 min at room temperature. Diaminobenzene was used as the chromogen and hematoxylin as the nuclear counterstain. Sections were dehydrated, cleared and mounted.

Evaluation of B7-H3 staining in cancer cells was evaluated by authorized pathologists who had no knowledge of the patients’ clinical status and outcome. B7-H3 expression scores were given separately for the stained area and for the intensity of staining. Quantification was made as follows; ≤33% of the cancer cells: 1, >33 to ≤66% of the cancer cells: 2, >66% of the cancer cells: 3; intensity of staining: absent/weak: 1, moderate: 2, strong: 3. The intensity of B7-H3 staining was considered weak when either cytoplasmic expression or rare membranous condensation was present, moderate when incomplete and discontinuous moderate membranous expression was present, and strong when complete membranous expression of the molecule was present. Each section had a final grade that derived from the multiplication of the area and intensity scores. Sections with a final score of ≤3 were classified as tumors with low B7-H3 expression, whereas sections with a final score of >3 were classified as tumors with high B7-H3 expression.

### B7-H3 siRNA transfection

To further analyze the role of B7-H3 in HCC malignancy, HepG-2 and SMMC7721 cells were tranfected with B7-H3 shRNA plasmid using Lipofectamine^2000^ (Invitrogen, CA). Each shRNA vector is cloned in pGFP-V-RS (Origene Technologies, Inc.) plasmid under U6 promoter for mammalian cell expression. The set sequence of the B7-H3 siRNA contains 5 vials of gene-specific siRNA expression vectors in pGFP-V-RS plasmid. We selected the most efficient one to carry out the following experiment. This sequence of the *B7-H3* siRNA is 5′-TGAAACACTCTGACAGCAAAGAAGATGAT-3′. The plasmid contains a non-effective siRNA cassette against green fluorescent protein as a scrambled negative control (Origene Technologies, Inc.) In brief, about 3 × 10^5^ cells were seeded per well in a 6 well plate. After 24 h, the cells were transfected with 1.5 μg of cDNA or siRNA plasmid for 6 h, and the media were replaced with fresh growth medium. At 48 h after transfection, cells were harvested for analysis. Transfection efficiency was evaluated by RT-PCR and western blotting assay, respectively.

### Cell proliferation by MTT assay

The MTT assay was used to study the effect of B7-H3 siRNA interference on HCC cell proliferation. Cells were seeded at a density of 5 × 10^3^ cells per well in 200 μl of DMEM medium into 96-well plates, and cultured overnight. At different time points, the medium was replaced with 100 μl fresh medium containing 0.5 mg/ml MTT (Sigma, USA). Four hours after the addition of MTT, the supernatants were removed and discarded. 150 μl of dimethylsulfoxide (DMSO, Sigma) was added to each well to dissolve the crystals. Cell viability was determined by scanning with a microplate reader at a wavelength of 490 nm. Each experiment was performed in triplicate and repeated at least three times.

### Apoptosis assay

The induction of apoptosis by B7-H3 siRNA transfection was evaluated with a Cell Death Detection ELISA^Plus^ kit (Roche, Germany) according to the manufacturer’s instruction. Cells were cultured in 96-well plates, starved by serum deprivation for 24 h. Then, cells were transfected with B7-H3 siRNA or scrambled non-target siRNA for 24, 48 and 72 h as indicated. Measurements were made using an ELISA reader at 405 nm. Each group was repeated in six wells and the results were averaged.

### Scratch wound healing assay

B7-H3 or control siRNA transfected HCC cells were preincubated with serum-free medium for 24 h, and cell layers were scraped with a pipette tip when cells reached 80% confluence. Cells were then washed twice with PBS and incubated in serum-free medium at 37C in a 5% CO_2_ incubator for 24 h. Photographs were taken at different times from 0 to 48 h. Wound width was measured at 200 × magnification using a BX50 microscope (Olympus®). Ten measurements were made at random intervals along the wound length. Data were averaged and expressed as the mean wound distances. This experiment was done in triplicate.

### Invasion assay

Invasion assays were done using transwell matrigel invasion chambers with a pore size of 8 μm coated with 1 μg/cm^2^ to 2 μg/cm^2^ martigel (BD, USA). Cells were detached and resuspended in serum-free DMEM. A total of 2 × 10^4^ cells in 100 μl of serum-free media were seeded onto the upper portion of a 24-well matrigel chamber. The lower compartment contained DMEM with 10% FBS. After incubation at 37°C for 24 h in a 5% CO_2_ atmosphere, the non-invading cells and gel were removed from the upper chamber with cotton tipped swabs. The cells were rinsed with PBS and cells on the filters were fixed with methanol for 30 min and stained with crystal violet solution. The number of invading cells on the filters was counted in 5 random fields per filter at 200× magnification in triplicate wells of each group.

### Western blotting

HCC cells were washed with PBS twice and lysed with 1 mL RIPA lysis buffer containing protease and phosphatase inhibitor for 30 min on ice. After removing the insoluble material by 12,000 × g centrifugation for 30 min at 4°C, the supernatants were collected. Cell lysate protein content was determined using a Bicinchoninic acid (BCA) protein assay kit. Equivalent amounts of whole cell extracts were subjected to SDS-PAGE and transferred to PVDF membranes. The membranes were blocked with 5% non-fat milk for 2 h and then incubated with respective primary antibody overnight at 4°C followed by the incubation with the appropriate HRP-conjugated secondary antibody for 2 h at room temperature. Blots were visualized with an ECL detection kit (Pierce, USA) and GAPDH was used as a loading control.

### Gelatin zymography

For MMP-2 and MMP-9 activity detection, cell lysate from treated cells were prepared and subjected to electrophoresis with 8-12% SDS polyacrylamide gels containing 0.1% gelatin. After electrophoresis, the gels were washed with 2.5% Triton X-100 for 30 min to remove SDS and incubated in a reaction buffer at 37°C for 16 h. Finally, the gels were stained with 0.5% Coomassie brilliant blue R-250 solution containing 10% acetic acid and 20% methanol for 30 min and then destained with 7.5% acetic acid solution containing 10% methanol. Areas of gelatinase activity appear as clear bands against the blue-stained gelatin background.

### Statistical analysis

The Mann–Whitney *U* test, *χ*2 test or Spearman rho test were performed for comparative statistical evaluations among groups and for correlation analysis with histological and clinical parameters (age, gender, tumor stage, tumor grade, and postoperative survival). The Kaplan-Meier method was used to analyze survival data and the log-rank test was taken to control differences in patients’ survival. Other data are shown as the mean and range or mean ± SD of 3 independent experiments. Statistical comparisons were performed using Student’s *t* test. All *p* values were determined by 2-sided tests with significance considered at *p* < 0.05 using SPSS 20.0 for Windows.

## Results

### Increased B7-H3 expression is significantly correlated with HCC metastasis

**Figure 1:**
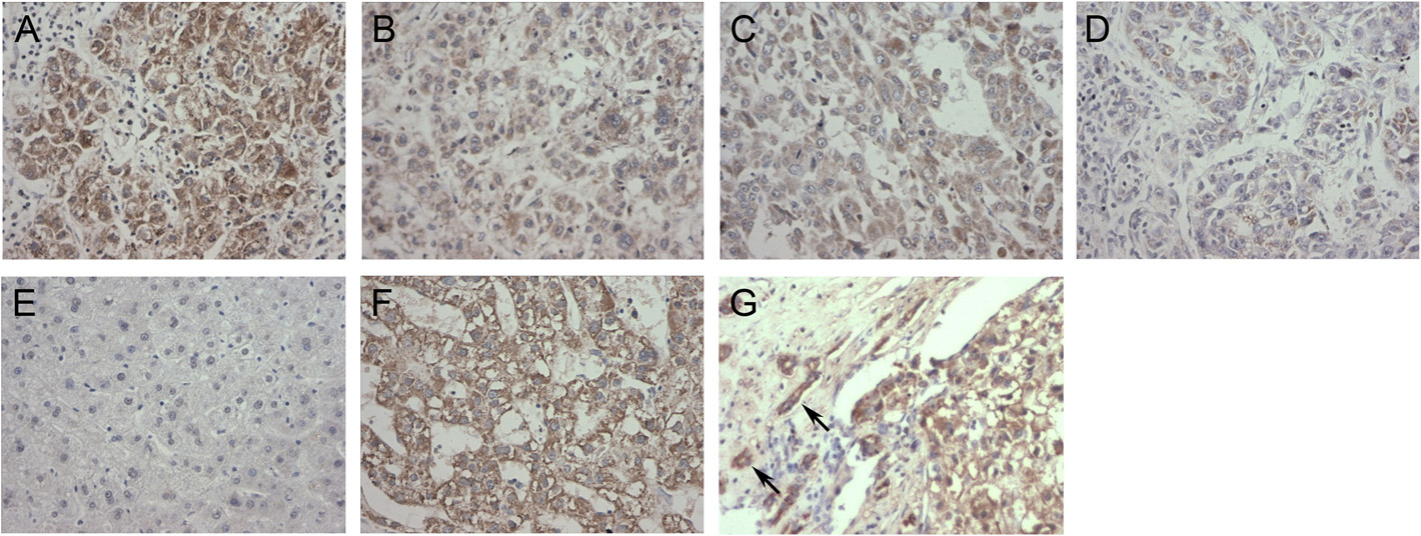
Representative immunohistochemical analysis of B7-H3 expression in tissue sections of HCC. B7-H3 immunostaining in HCC tissues **(A)** Strong positive, **(B)** Moderate positive, **(C)** Weak positive. B7-H3 immunostaining in HCC adjacent liver tissues **(D)**, normal liver tissues **(E)** and metastatic tumor **(F)**. Higher B7-H3 expression was often observed in tumor cells at the tumor edge **(G)**, invading (arrows) to surrounding tissue, ×200 magnification.

Among the examined 116 HCC tissues, 109 showed positive B7-H3 protein expression in tumor cells. In our case series, the expression of B7-H3 was variably positive in HCC tumor cells and weak and focal positive or negative in normal hepatic tissue (Figure 1A-F). A total of 79.3% of tumor samples were identified as high B7-H3 staining, while 20.1% showed a low degree of B7-H3 staining. High expression of B7-H3 correlated significantly with late TNM stage (*p* = 0.038), the presence of vascular invasion (*p* = 0.001), lymph metastasis (*p* = 0.041), and the formation of microsatellite tumors (*p* = 0.016), which is considered evidence of intrahepatic tumor metastasis (Table 1). Interestingly, increased expression of B7-H3 was often observed in tumor cells at the edge of the tumor and in cells invading to surrounding tissue (Figure 1G).

**Table 1:** **Relationship between B7-H3 expression and clinico-pathological parameters in HCC**

**Index**	**Case**	**B7-H3 expression**		
		**Low**	**High**	***p***
**Age (y)**				
≤50	43	9	34	0.570
>50	73	15	58	
**Gender**				
Male	84	16	68	0.320
Female	32	8	24	
**Vascular Invasion**				
+	50	3	47	**0.001**
-	66	21	45	
**TNM stage**				
I-II	20	57	77	**0.038**
III-IV	4	35	39	
**Lymph metastasis**				
+	32	3	29	**0.041**
-	84	21	63	
**Tumor size (cm)**				
≤5	77	21	56	**0.010**
>5	39	3	36	
**Tumor encapsulation**				
+	18	1	17	0.071
-	98	23	75	
**Microsatellite tumors**				
Yes	31	2	29	**0.016**
No	85	22	63	
**Child-Pugh classification**				
A	60	13	47	0.485
B	56	11	45	
**Liver cirrhosis**				
weak	24	8	16	0.203
moderate	58	10	48	
strong	34	6	28	
**HBsAg**				
+	76	13	63	0.142
-	40	11	29	
**AFP**				
≤200	48	8	40	0.242
>200	67	16	51	
**Transfusion**				
+	47	8	39	0.286
-	69	16	53	

Values in bold signify *p* < 0.05.

To investigate the correlation between B7-H3 overexpression and HCC metastasis, the expression level of B7-H3 was analyzed in 32 pairs of HCCs samples including 32 primary HCCs patients without metastasis and 32 primary metastatic HCCs patients at first diagnosis. Increased intensity of tumor B7-H3 staining was detected more significantly in metastatic HCC tumors (*p* = 0.019, Wilcoxon signed rank test; Table 2). These data further support the hypothesis that B7-H3 plays an important role in HCC metastasis.

**Table 2:** **B7-H3 intensity in 32 pairs of primary HCCs and matched metastatic HCCs**

	**B7-H3 intensity of matched metastatic HCCs**				**Total**
		**weak**	**moderate**	**strong**	
**B7-H3 intensity of primary HCCs**	**weak**	1	3	2	6 (18.8%)
	**moderate**	0	7	5	12 (37.4%)
	**strong**	0	2	12	14 (43.8%)
**Total**		1 (0.04%)	12 (37.5%)	19 (59.4%)	32

### Increased B7-H3 expression is predictive of poor survival in HCC patients

**Figure 2:**
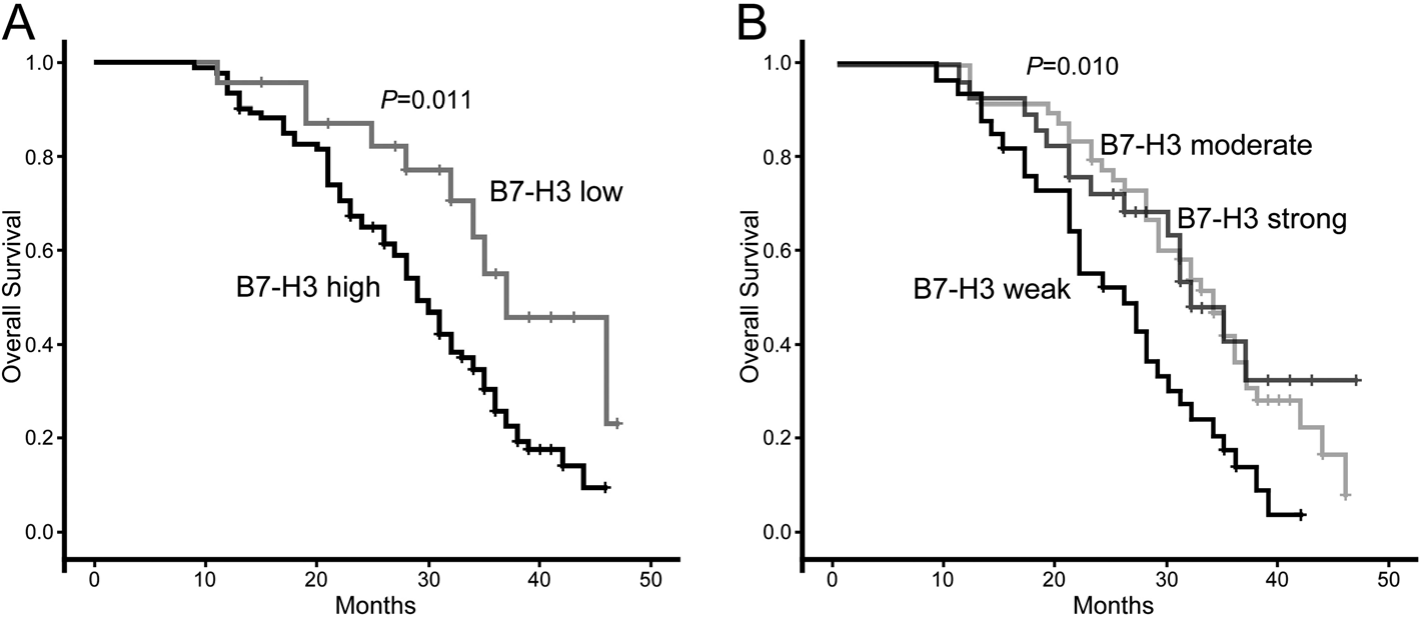
Kaplan-Meier overall survival curve of HCC patients in correlation with B7-H3 expression **(A)** and its intensity level **(B)**.

Of the 116 patients with HCC who underwent hepatic resection, 79 (68.1%) finally died. We analyzed the prognostic significance by comparing carcinomas with high B7-H3 levels to those with low B7-H3 expression. The low-B7-H3 group showed significantly better overall survival than the high-B7-H3 group (log-rank test, *p* = 0.011). A Kaplan–Meier curve regarding the association between B7-H3 expression in tumor cell and overall survival is shown in Figure 2A. The median survival in patients with low tumor B7-H3 expression was 37.1 ± 4.62 months and 29.2 ± 1.27 months in patients with high B7-H3 expression. After univariate analysis, HCC patients with high-B7-H3 tumor cells were more likely to die compared with counterparts with low-B7-H3 tumors (risk ratio, 2.275; 95% CI, 1.166-4.438; *p* = 0.016). After adjustment for the TNM stage, vascular invasion and lymph metastasis, patients with high-B7-H3 tumor cells remained significantly more likely to die from HCC compared with patients with low-B7-H3 tumors (risk ratio, 2.586; 95% CI, 1.271-5.260; *p* = 0.009). In addition, B7-H3 staining intensity was also associated with HCC patients’ survival. Cases with a strong or moderate B7-H3 staining intensity in HCC showed a significantly shortened mean survival time compared to the cases with weaker staining (log-rank test, *p* = 0.010) (Figure 2B). Therefore, these data suggested that B7-H3 expression might be functionally important in tumor progression of HCC, and high tumor B7-H3 expression predicts poor prognosis for HCC patients.

### Silenced B7-H3 expression has no obvious effect on HCC cell proliferation and apoptosis

Cultured HCC cell lines (HepG2 and SMMC7721) all constitutively expressed B7-H3 at both mRNA and protein level. RT-PCR results showed that there was markedly decreased gene expression after silencing B7-H3 for 24 h compared with the control groups. Accordingly, a similar decrease was also found in protein synthesis and secretion after 48 h in B7-H3 knockdown cells (Additional file 1: Figure S1).

**Figure 3:**
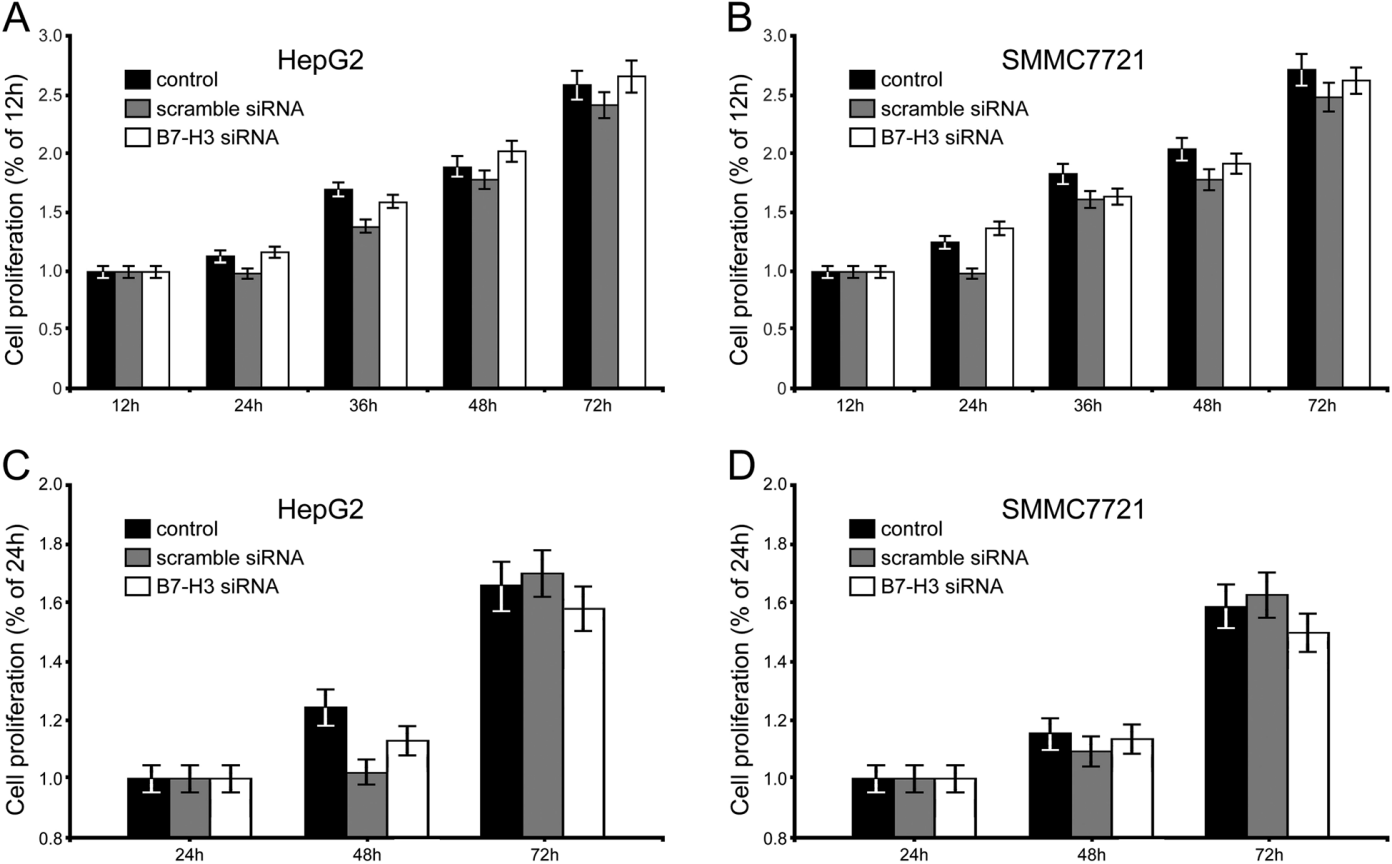
B7-H3 siRNA interference effects on HepG2 and SMMC7721 cell proliferation **(A-B)** and apoptosis **(C-D)**. Data represent mean ± SEM of three independent experiments.

To characterize the role of B7-H3 in HCC cell growth, we measured cell proliferation rate in vitro by MTT assay. Results showed that there was no statistical significance in cellular proliferation between the parental, scramble siRNA-transfected and B7-H3 silenced cells (Figure 3A-B). Besides, apoptotic ELISA assay revealed that downregulation of B7-H3 did not increase cell apoptosis, compared with the other two control cells (Figure 3C-D).

### Silenced B7-H3 expression suppresses scratch wound healing ability of HCC cells

**Figure 4:**
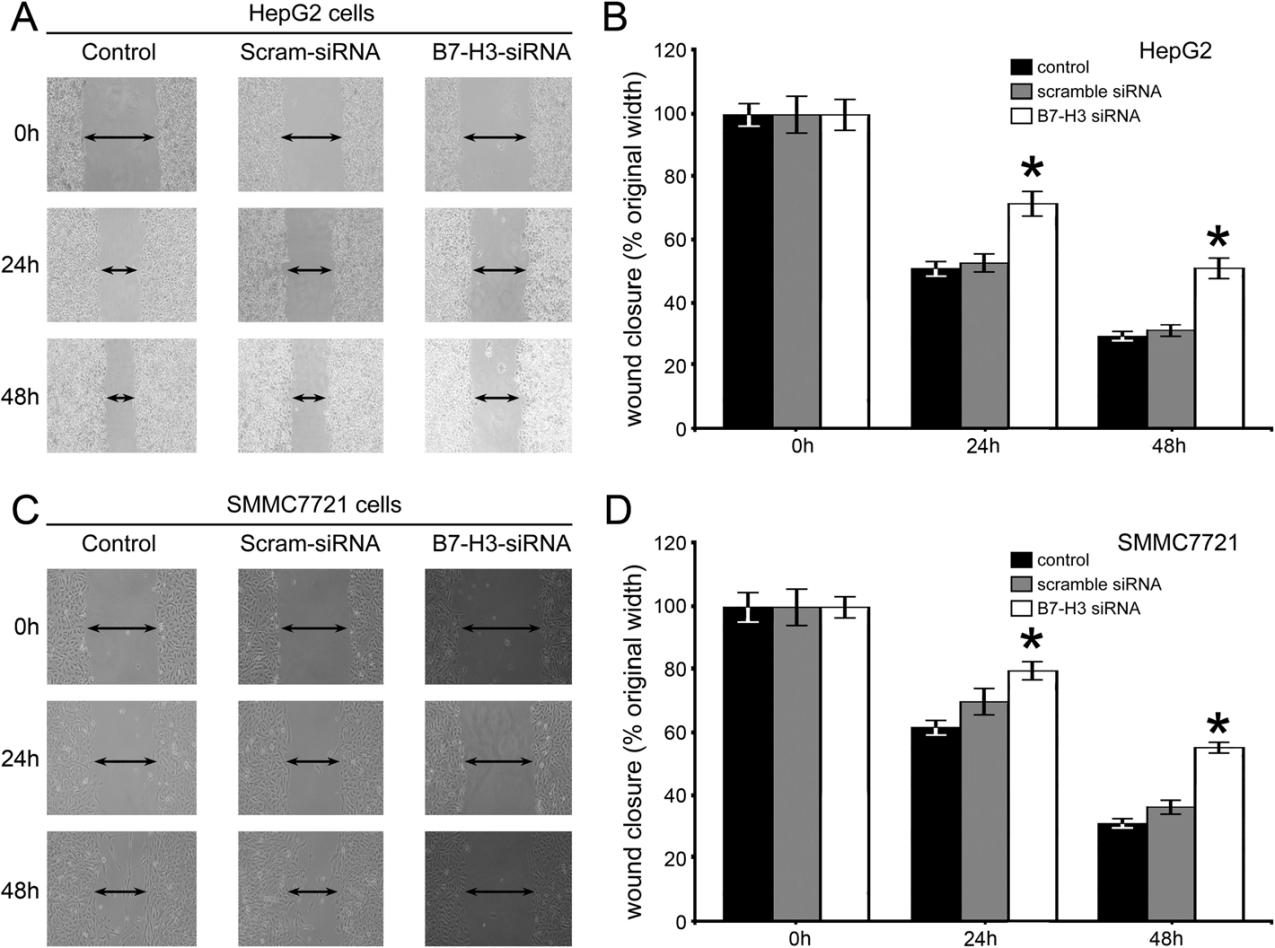
B7-H3 siRNA interference effects on HepG2 **(A-B)** and SMMC7721 **(C-D)** cell migration by wound healing analysis. The confluent cells were wounded by sterile pipettes and the status of wound closure were observed and photographed after 48 h. All the experiments were repeated for three times (magnification, 200×).

We next examined the cytological effect of B7-H3 on movement ability of HCC cells by scratch healing assay. Results showed that depletion of B7-H3 in HepG2 and SMMC7721 cell lines could not contribute to healing the wound 48 h after scratching, in contrast to the parental cells and empty vector-transfected cells which were significantly more efficient in healing, as indicated by the mean wound distances (Figure 4). To further confirm the function of B7-H3 in HCC cells, HepG2 cells were cultured in the presence or absence of anti-B7-H3 mAbs (10 μg/ml). Results showed that the cells in the control group migrated faster than those in the B7-H3 blockade group (Additional file 2: Figure S2A).

### Silenced B7-H3 expression suppresses invasion of HCC cells

**Figure 5:**
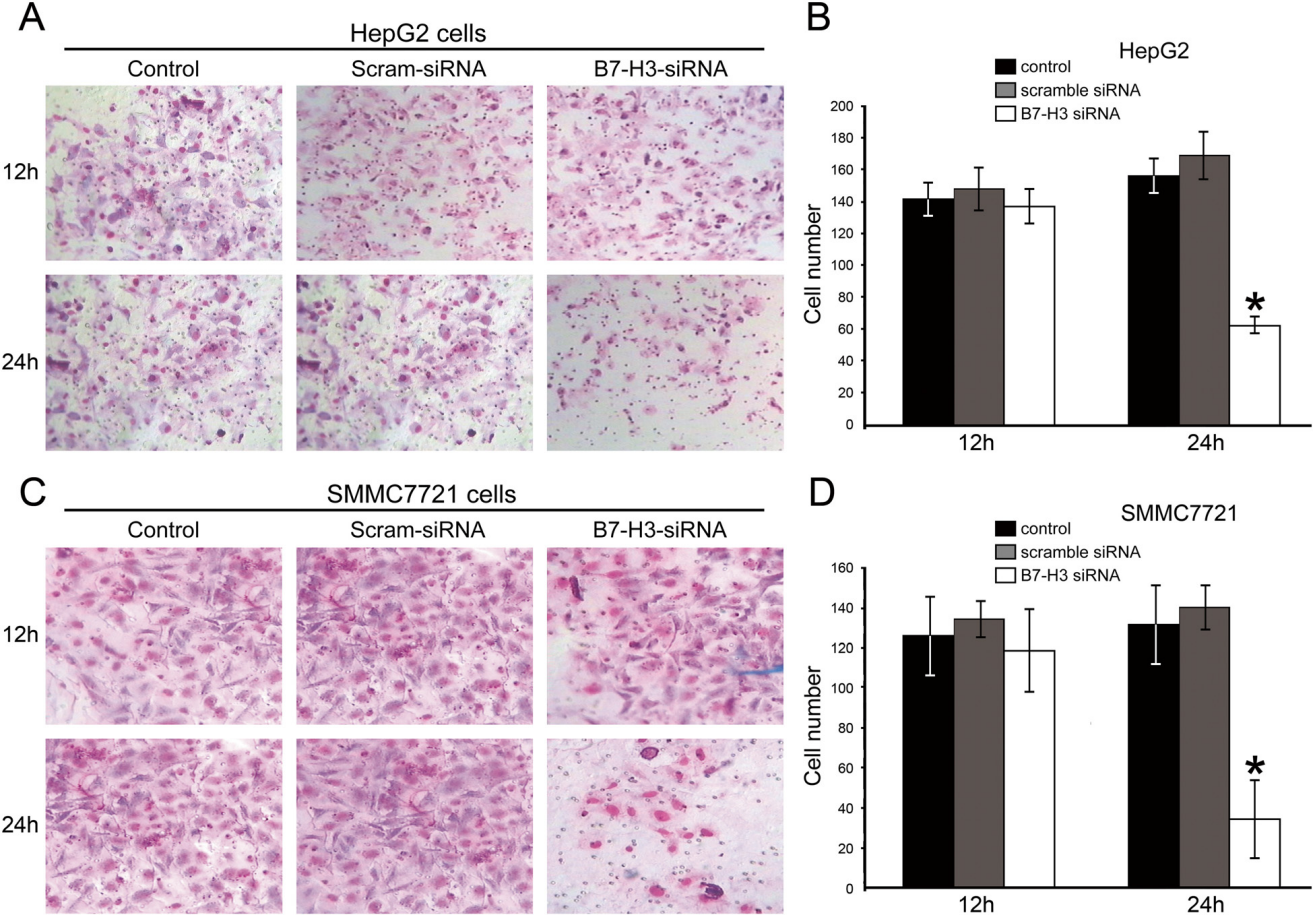
B7-H3 siRNA interference effects on HepG2 **(A-B)** and SMMC7721 **(C-D)** cell invasion by transwell chamber assay. Representative photographs of invasive HepG2 and SMMC7721 cells on the membrane, all the experiments were repeated for three times (magnification, 200×).

In order to further assess the influence of B7-H3 on HCC cells’ invasion, we employed cell transwell invasion assays to determine the key factor of malignant progression and metastasis upon B7-H3 downregulation. As shown in result, downregulation of B7-H3 led to significantly suppressed invasive ability of both HepG2 and SMMC-7721 cells (Figure 5). Number of invasive HepG2 B7-H3 knockdown cells is significantly lower compared to the control groups after 24 h (82.17 ± 7.00 vs 243.26 ± 14.07 and 208.37 ± 23.98, *p* < 0.05). Similarly, number of invasive SMMC-7721 B7-H3 knockdown cells is significantly lower compared to the control groups after 24 h (53.08 ± 6.16 vs 261.12 ± 11.07 and 283.52 ± 17.13, *p* < 0.05). The similar results were also observed in HCC cells after in the presence or absence of anti-B7-H3 mAbs (Additional file 2: Figure S2B). These results displayed an enhanced effect of B7-H3 on hepatocellular carcinoma cells invasion.

### Silenced B7-H3 expression suppresses migration and invasion of HCC cells by targeting EMT via JAK2/Stat3/slug signaling pathway

**Figure 6:**
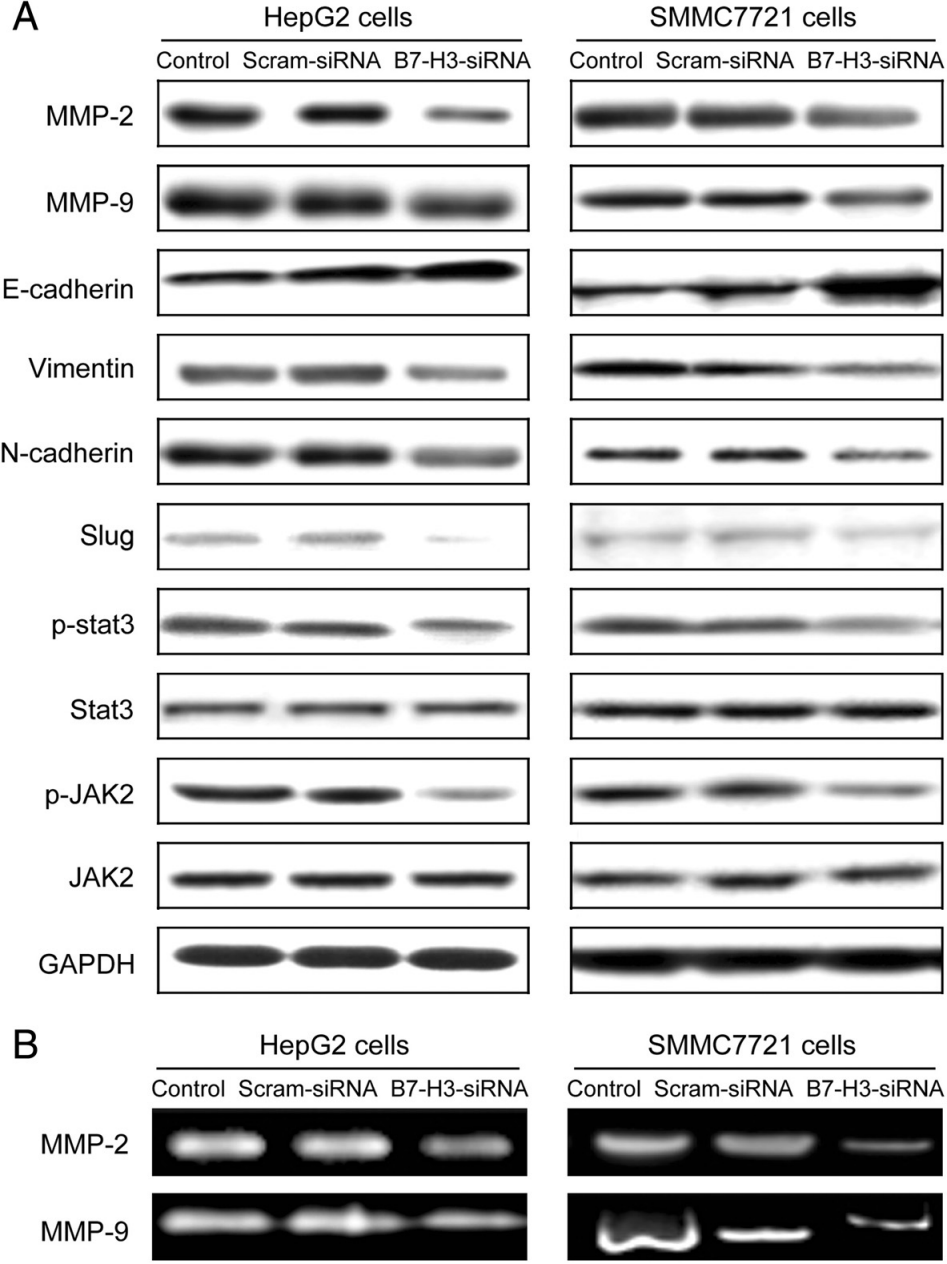
Western blot analysis for protein levels of MMP-2, MMP-9, E-cadherin, Vimentin, N- cadherin, Slug, p-stat3, stat-3, p-JAK2 and JAK2 in hepatocellular carcinoma cell lines HepG2 and SMMC7721. Protein levels were expressed as the ratio of target protein over GAPDH. All the experiments were repeated for three times **(A)**. Zymography experiments for detection of the activity of MMP-2 and MMP-9 in hepatocellular carcinoma cell lines HepG2 and SMMC7721 **(B)**.

Results showed loss of B7-H3 decreased the expression and activity of matrix metalloproteinase-2 (MMP-2) and MMP-9 significantly in both B7-H3-siRNA transfected HCC cell lines (Figure 6). In addition, B7-H3 is also involved in EMT process of HCC cells. After knockdown of B7-H3, the expressions of E-cadherin was up-regulated, while N-cadherin and Vimentin were down-regulated in each B7-H3 silenced groups compared with the negative control group (Figure 6). These results suggested that B7-H3 may play an important role in EMT in human hepatocellular carcinoma.

JAK/Stat pathway is critical for cytokine and growth factor-mediated responses regulating EMT biology in fibrogenesis and cancer [17]. In the light of the evidence supporting the activated status of Stat3 in HCC [18] and the role of Stat3 in cell motility and EMT [19], this study sought to directly examine the role of this pathway in B7-H3-induced EMT in HCC cells. After treatment of B7-H3 siRNA, the protein level of p-sata3 and p-JAK2 was dramatically reduced in HepG2 and SMMC7721 cells transfected with B7-H3 siRNA plasmids, while no influence on the total expression of Stat3 and JAK2, compared with control shRNA plasmids (Figure 6). In addition, EMT-related transcription factors Slug was downregulated after B7-H3 silenced (Figure 6), whereas Snail seemed to be not significantly affected. The similar results were also observed in HCC cells after in the presence or absence of anti-B7-H3 mAbs (Additional file 2: Figure S2C-D). This result could be the explanation that B7-H3 may regulate EMT process of HCC via partially activating JAK2/ Stat3/Slug signaling pathway.

## Discussion

Although curative surgery and chemotherapy offer an opportunity for HCC patients, the cumulative 5-year recurrence rate still ranges from 40% to 80% because of high potential for vascular invasion and metastasis [20,21]. Consequently, the identification of certain cell surface molecule that is uniformly and stably expressed by primary and metastatic HCC encompasses a vital finding and an opportunity to improve HCC treatment. This is especially true when the function of the molecule is relatively well defined and can be readily linked to mechanisms for cancer progression. In the present study, B7-H3 was more frequently elevated in HCC tissue with late TNM staging, vascular and lymph node involvement and tumor invasion. Higher expression level of B7-H3 was often detected in tumor cells invading into the surrounding tissue and blood vessel. Moreover, the invasive and metastatic effect of B7-H3 in clinical specimens was also addressed by IHC containing in primary metastatic HCCs’ samples. Increased B7-H3 intensity level was detected in 59.4% of the metastatic HCC tumors compared other non-metastatic primary HCCs. These results suggest that HCC is likely to become aggressive and metastatic when B7-H3 expression is high, suggesting that B7-H3 plays an important role in tumor invasion of HCC. Furthermore, we also found that patients with high B7-H3 expression or strong B7-H3 intensity level experienced significantly shorter periods of postoperative survival. These findings directly demonstrate the clinical significance of B7-H3 to help tumor cell escaping form immunosurvillance and further invasion and metastasis in HCC.

B7-H3 is a new member of B7 family that has been implicated as a potential regulator of antitumor response [22]. The role of B7-H3 in tumor has been widely studied and its function has been associated with the regulation of immune responses [13,16,23]. However, recent work by Nygren et al. group indicates that B7-H3 also has non-immunological effects on invasion and chemoresistance in human cancers [24]. Based on the previous results we collected, it has reason to believe that B7-H3 might be involved in HCC cells’ biological process, which in turn stimulated the malignant potency of HCC cells and establish a vicious cycle of mutually reinforcing mechanisms to sustain the activity and promotes the malignant behavior of HCC. Therefore, we silenced B7-H3 in HepG2 and SMMC7721 cells to explore the effects of B7-H3 in HCC cells’ biology and its underlying mechanism. Results showed that a transient knockdown of B7-H3 significantly suppressed the migratory and invasive ability of HCC cells in vitro. This suggests that B7-H3 has a putatively important role in tumor migration and invasiveness, indicating higher aggressiveness and poor clinical outcome. Many data have revealed that cell proliferation affected the outcomes of both transwell assay and wound healing assay [25], it is essential to examine whether B7-H3 knockdown affected the proliferation of HepG2 and SMMC7721 cells. Results showed that the proliferation of B7-H3 siRNA transfected cells was no obvious difference from that of controls. Besides, cell apoptosis was also not altered significantly between B7-H3 siRNA group and controls. Taken together, these results suggested that knockdown of B7-H3 decreased the invasion and metastasis of HCC cells and this inhibitory effect was not dependent on the proliferation of tumor cells.

MMPs are a family of related zinc-dependent proteinases that are believed to play important role in the invasive process and metastasis of cancer cells [26]. Among them, MMP-2 and MMP-9 are the most concerned and their functions have been well-characterized in HCC [27,28]. Our previous study revealed that B7-H3 regulated invasion of osteosarcoma cells at least partly through MMP-2 [29]. Tekle et al. also reported that B7-H3 contributed to the metastatic capacity of melanoma cells by modulation of MMP-2 and signal transducer and activator of Stat3 [30]. In this present study, we show that B7-H3 depletion could lead to the decreased expression and activity of MMP-2 and MMP-9 in HCC cells, indicating that a decreased metalloproteinase activity in the B7-H3 knockdown cells, which at least partly might explain the reduced invasive capacity of these cells.

Metastasis generally involves shedding of tumor cells from the primary sites and their infiltration into peripheral tissues, which is caused by various factors, where intercellular adhesion is reduced and thus tumor cells adhere to the basal membrane and then move out of the primary site as the extracellular matrix is degraded [31,32]. The EMT is a key event for cancerous cells to acquire the capability of migration and invasion of HCC cells [33]. These processes are tightly regulated by temporally and spatially regulated expression and activation of many signal molecules [17,34]. To better elucidate the invasive and metastatic mechanisms of B7-H3, the effect of B7-H3 depletion on the EMT was investigated. As expected, the epithelial markers E-cadherin were up-regulated, whereas the mesenchymal markers N-cadherin and vimentin were downregulated in B7-H3 siRNA-tranfected cells. Moreover, we observed that the EMT-related transcription factors Slug was downregulated after B7-H3 silenced and phosphorylation levels of Stat3 and JAK2 were decreased. All of these results clearly demonstrated that the EMT induced by B7-H3 partially via JAK2/Stat3/Slug signaling pathway is an important mechanism underlying HCC development and metastasis. Furthermore, this could be the reason why B7-H3 overexpression is correlated with higher incidence of intrahepatic metastasis and recurrence in immunohistochemistry.

To conclude, the overexpression of B7-H3 in HCC creates a milieu of pro-metastatic factors that may play a role in very early and/or reactivated intrahepatic metastasis. A mechanism by which B7-H3 knockdown inhibits the EMT process and JAK2/STAT3/Slug signaling pathway plays important role in this process. This finding supports the possibility of B7-H3 and/or its associated molecules as targets for anti-metastatic therapy in HCC.

## Additional files

Additional file 1: Figure S1. RT-PCR and western blot detection of B7-H3 gene expression in hepatocellular carcinoma cell lines HepG2 and SMMC7721 after siRNA interference. GAPDH was used as an internal control. All the experiments were repeated for three times. Additional file 2: Figure S2. The migratory ability was detected by wound healing assay in HepG2 cells treated with B7-H3 blocking antibody or control (A). The invasive ability was detected by transwell chamber assay in HepG2 cells treated with B7-H3 blocking antibody or control (B). Western blot analysis for protein levels of MMP-2, MMP-9, E-cadherin, Vimentin, N- cadherin, Slug, p-stat3, stat-3, p-JAK2 and JAK2 in hepatocellular carcinoma cell line HepG2 treated with B7-H3 blocking antibody or control (C). Zymography experiments for detection of the activity of MMP-2 and MMP-9 in hepatocellular carcinoma cell line HepG2 treated with B7-H3 blocking antibody or control (D).
