# Correction to: B7-H3 promotes aggression and invasion of hepatocellular carcinoma by targeting epithelial-to-mesenchymal transition via JAK2/STAT3/Slug signaling pathway

**DOI:** 10.1186/s12935-021-02216-z

**Published:** 2021-10-28

**Authors:** Fu-biao Kang, Ling Wang, Heng-chuan Jia, Dong Li, Hai-jun Li, Yin-ge Zhang, Dian-xing Sun

**Affiliations:** 1 Department of Liver Diseases, Bethune International Peace Hospital, Shijiazhuang, Hebei People’s Republic of China; 2 Chinese PLA Medical School, Chinese PLA General Hospital, Beijing, People’s Republic of China; 3 Cancer Research Institute, The Fourth Hospital of Hebei Medical University, Shijiazhuang, Hebei People’s Republic of China

## Correction to: Cancer Cell Int (2015) 22:607 https://doi.org/10.1186/s12935-015-0195-z

Following the publication of the original article [1], we were notified of an error in Fig. 5.

Both incorrect and corrected Fig. 5 are presented in this erratum. The revision does not affect the results and conclusions of the article.

Originally published Fig. 5.


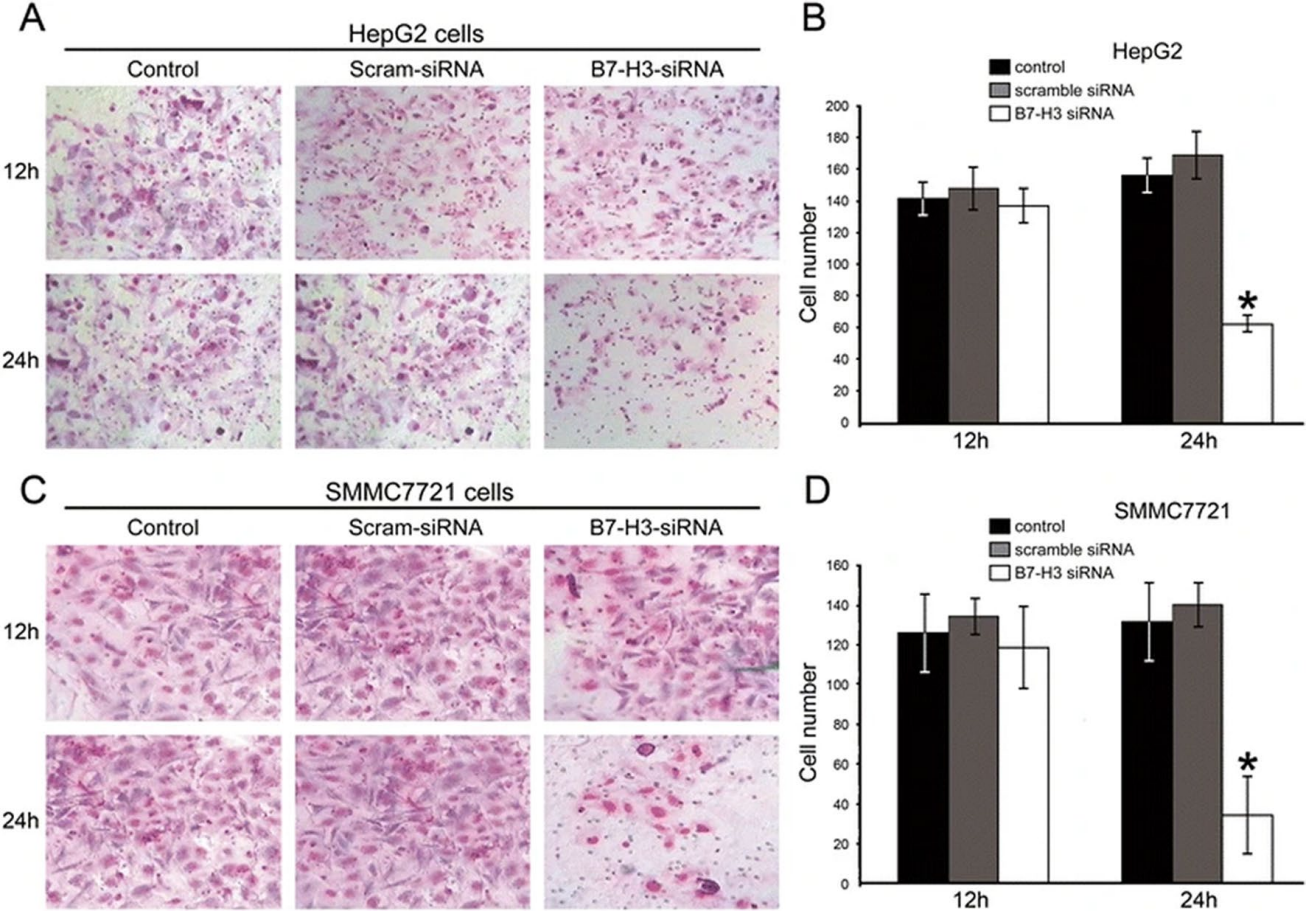


B7-H3 siRNA interference effects on HepG2 (**A**-**B**) and SMMC7721 (**C**-**D**) cell invasion by transwell chamber assay. Representative photographs of invasive HepG2 and SMMC7721 cells on the membrane, all the experiments were repeated for three times (magnification, 200×)

Corrected Fig. 5.


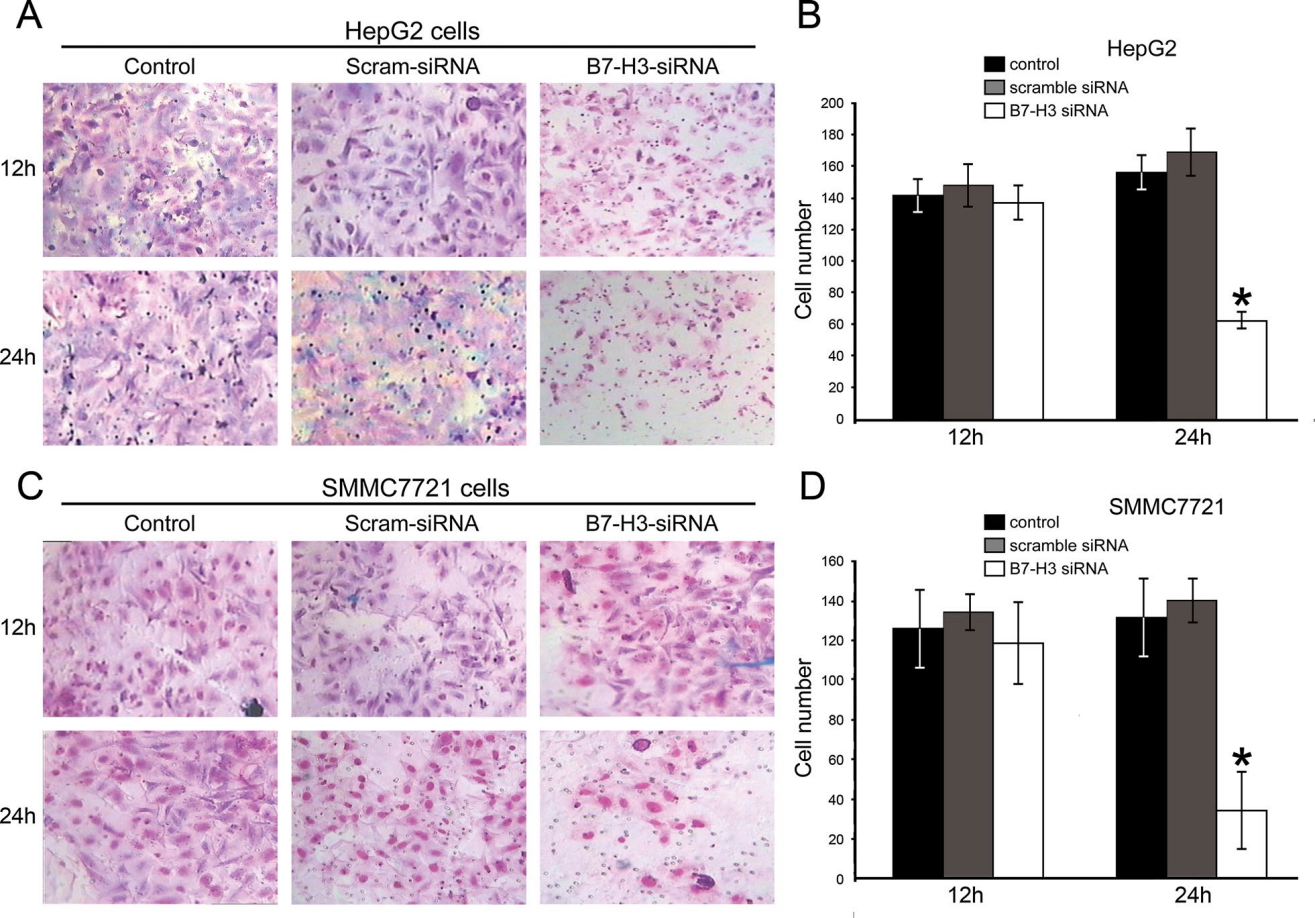


B7-H3 siRNA interference effects on HepG2 (**A**, **B**) and SMMC7721 (**C**, **D**) cell invasion by transwell chamber assay. Representative photographs of invasive HepG2 and SMMC7721 cells on the membrane, all the experiments were repeated for three times (magnification, × 200)

The original article has been corrected.
