# Supplementary figures and images for: B7-H3 promotes aggression and invasion of hepatocellular carcinoma by targeting epithelial-to-mesenchymal transition via JAK2/STAT3/Slug signaling pathway
Source: Cancer Cell Int. 2015 Apr 21;15:45. doi: 10.1186/s12935-015-0195-z (PMC4407415; doi:10.1186/s12935-015-0195-z)

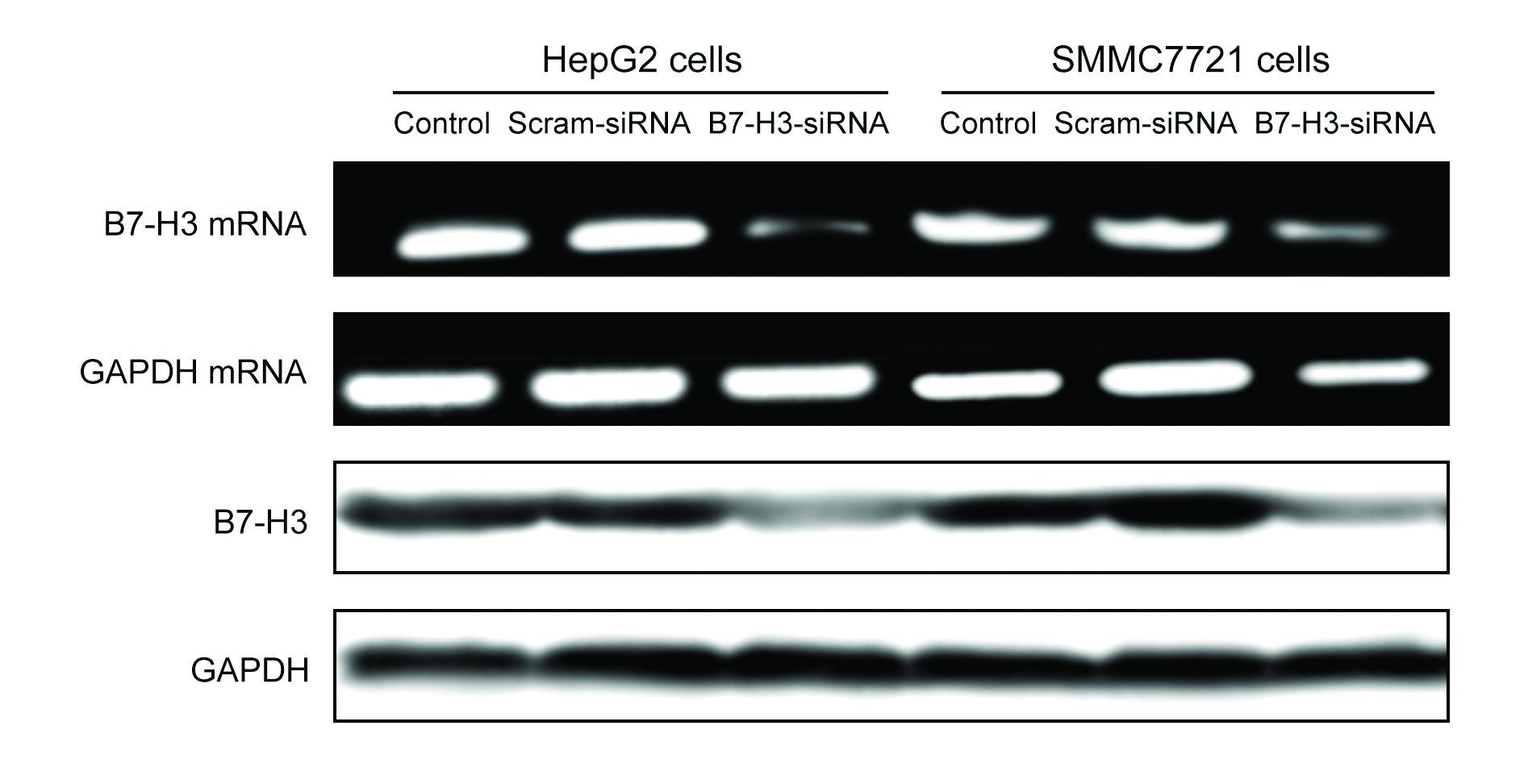

Supplement: Additional file 1: Figure S1. — RT-PCR and western blot detection of B7-H3 gene expression in hepatocellular carcinoma cell lines HepG2 and SMMC7721 after siRNA interference. GAPDH was used as an internal control. All the experiments were repeated for three times. [file 12935_2015_195_MOESM1_ESM.jpeg]

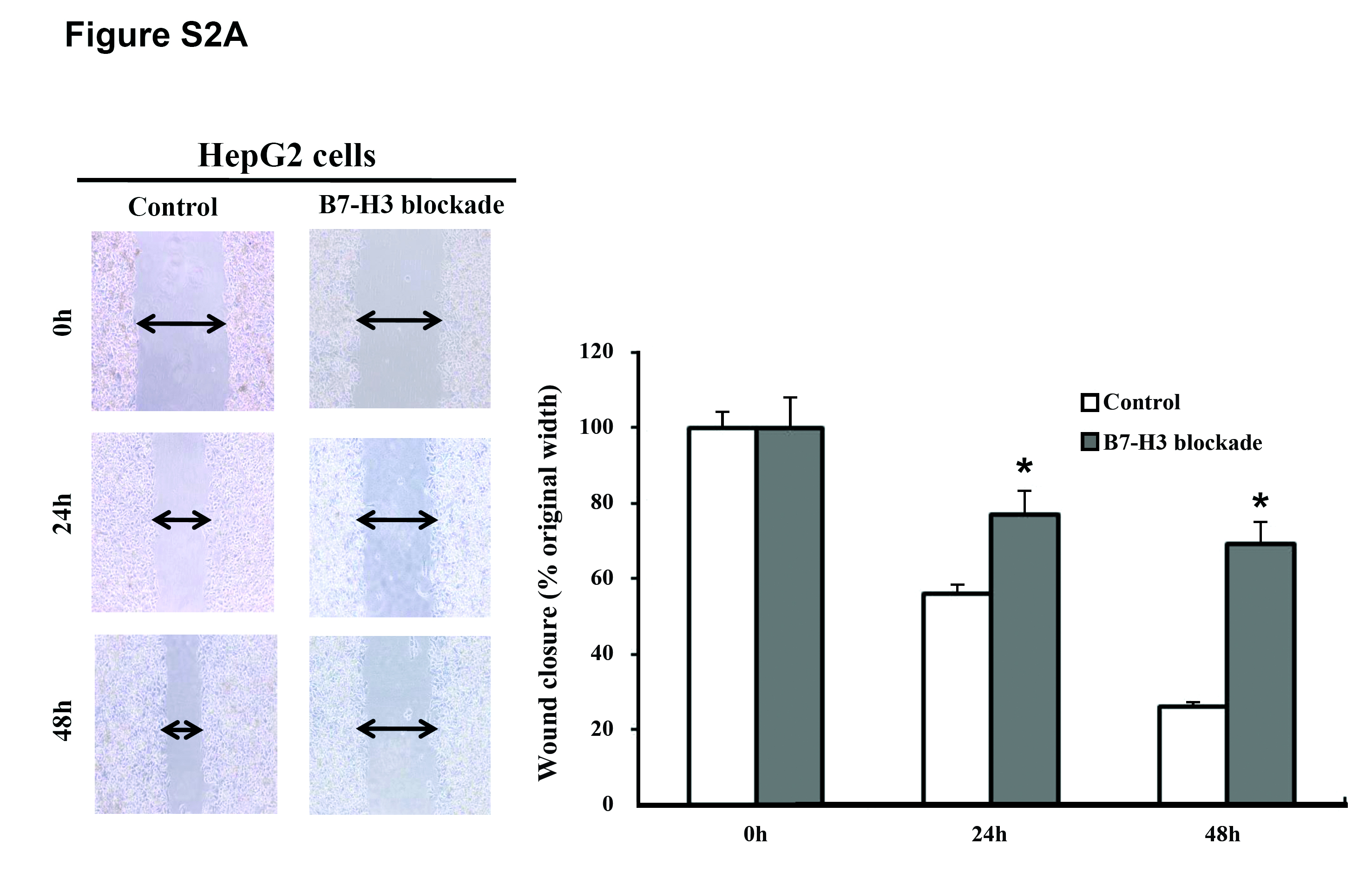

Supplement: Additional file 2: Figure S2. — The migratory ability was detected by wound healing assay in HepG2 cells treated with B7-H3 blocking antibody or control (A). The invasive ability was detected by transwell chamber assay in HepG2 cells treated with B7-H3 blocking antibody or control (B). Western blot analysis for protein levels of MMP-2, MMP-9, E-cadherin, Vimentin, N- cadherin, Slug, p-stat3, stat-3, p-JAK2 and JAK2 in hepatocellular carcinoma cell line HepG2 treated with B7-H3 blocking antibody or control (C). Zymography experiments for detection of the activity of MMP-2 and MMP-9 in hepatocellular carcinoma cell line HepG2 treated with B7-H3 blocking antibody or control (D). [file 12935_2015_195_MOESM2_ESM.zip › Additional file 2/12935_2015_195_MOESM2_ESM.tiff]

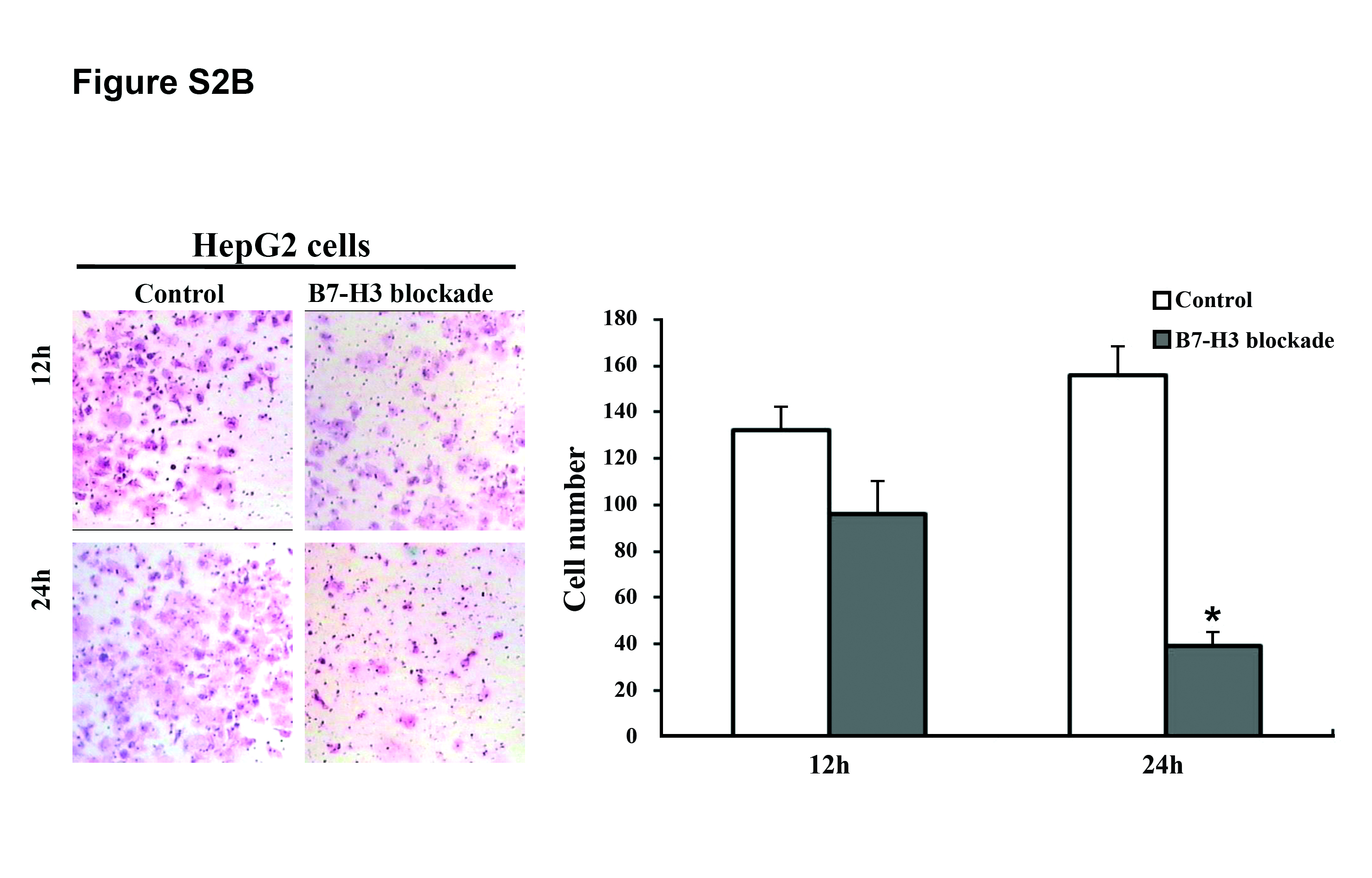

Supplement: Additional file 2: Figure S2. — The migratory ability was detected by wound healing assay in HepG2 cells treated with B7-H3 blocking antibody or control (A). The invasive ability was detected by transwell chamber assay in HepG2 cells treated with B7-H3 blocking antibody or control (B). Western blot analysis for protein levels of MMP-2, MMP-9, E-cadherin, Vimentin, N- cadherin, Slug, p-stat3, stat-3, p-JAK2 and JAK2 in hepatocellular carcinoma cell line HepG2 treated with B7-H3 blocking antibody or control (C). Zymography experiments for detection of the activity of MMP-2 and MMP-9 in hepatocellular carcinoma cell line HepG2 treated with B7-H3 blocking antibody or control (D). [file 12935_2015_195_MOESM2_ESM.zip › Additional file 2/12935_2015_195_MOESM3_ESM.tiff]

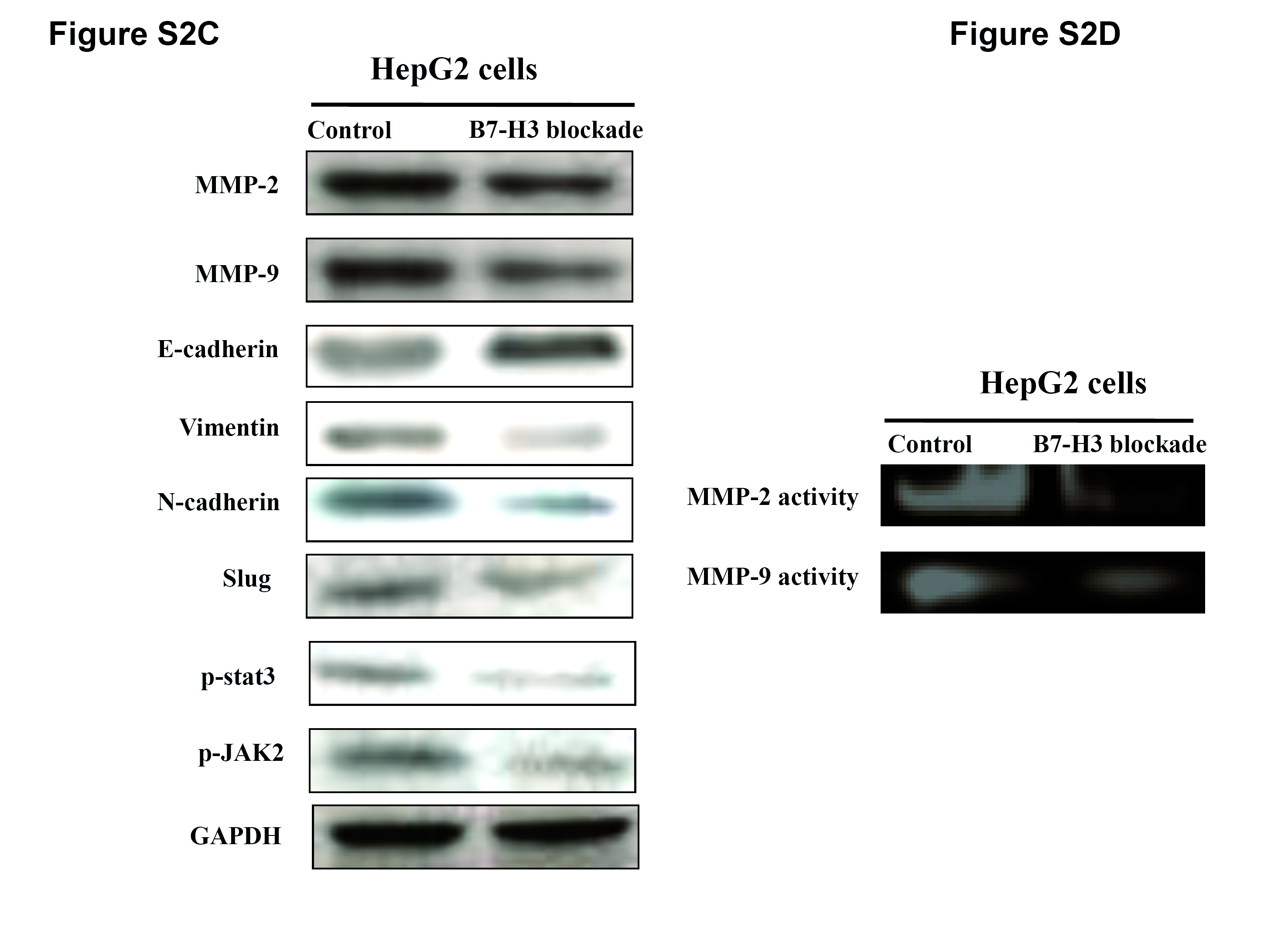

Supplement: Additional file 2: Figure S2. — The migratory ability was detected by wound healing assay in HepG2 cells treated with B7-H3 blocking antibody or control (A). The invasive ability was detected by transwell chamber assay in HepG2 cells treated with B7-H3 blocking antibody or control (B). Western blot analysis for protein levels of MMP-2, MMP-9, E-cadherin, Vimentin, N- cadherin, Slug, p-stat3, stat-3, p-JAK2 and JAK2 in hepatocellular carcinoma cell line HepG2 treated with B7-H3 blocking antibody or control (C). Zymography experiments for detection of the activity of MMP-2 and MMP-9 in hepatocellular carcinoma cell line HepG2 treated with B7-H3 blocking antibody or control (D). [file 12935_2015_195_MOESM2_ESM.zip › Additional file 2/12935_2015_195_MOESM4_ESM.tiff]
